# Supplementary material for: Current Situation and Perspectives on Hantaviruses in Mexico
Source: Viruses. 2019 Jul 12;11(7):642. doi: 10.3390/v11070642 (PMC6669582; doi:10.3390/v11070642)
Supplement: Supplementary file 1 [file viruses-11-00642-s001.pdf]

Supplementary table S1. Data analyzed by report

Reference	Report	State	Species	Analyzed	Positives	Serological method	Antigen
Hjelle, B., Anderson, B., Torrez-Martinez, N., Song, W., Gannon, W. L., & Yates, T. L. (1995). Prevalence and geographic genetic variation of hantaviruses of New World havest mice (*Reithrodontomys*): identification of a divergent genotype from a Costa Rican *Reithrodontomys mexicanus*. Virology, 207(2), 452–9. http://doi.org/10.1006/viro.1995.1104	Article	Oaxaca	*Reithrodontomis megalotis*	6	0	ELISA	SNV
Mantooth, S. J., Milazzo, M. L., Bradley, R. D., Hice, C. L., Ceballos, G., Tesh, R. B., & Fulhorst, C. F. (2001). Geographical distribution of rodent-associated hantaviruses in Texas. Journal of Vector Ecology, 26(1), 7–14.	Article	México	*Peromyscus hylocetes*	8	2	ELISA	CADV
			Peromyscus melanotis	9	2		
		Coahuila	*Peromyscus eremicus*	1	0	ELISA	CADV
			Dipodomys merriami	2	0		
Suzán, G., Ceballos, G., Mills, J., Ksiazek, T. G., & Yates, T. (2001). Serologic evidence of hantavirus infection in sigmodontine rodents in Mexico. Journal of Wildlife Diseases, 37(2), 391–393. http://doi.org/10.7589/0090-3558-37.2.391	Article	Mexico City	*Peromyscus maniculatus*	8	1	ELISA	SNV
			Microtus mexicanus	1	0		
			Reithrodontomys sumichrasti	1	1		
		Jalisco	*Baiomys musculus*	2	0		
			Osgoodomys banderanus	6	0		
			Oryzomys palustris	1	0		
			Liomys pictus	14	0		
Vado-Solís, I., Pérez-Osorio, C., Lara-Lara, J., Ruiz-Piña, H. a, Cárdenas-Marrufo, M., Milazzo, M. L., … Zavala-Velázquez, J. (2003). Evidencia serológica de infección por Hantavirus en población humana del estado de Yucatán, México. Revista de Biomedicina, 14(4), 221–225. Retrieved from http://www.uady.mx/sitios/biomedic/revbiomed/pdf/rb031442.pdf	Article	Yucatán	*Oligoryzomys fulvescens*	1	0	ELISA	SNV
			Peromyscus yucatanicus	69	0		
			Rattus rattus	12	0		
			Reithrodontomys gracilis	2	0		
			Sigmodon hispidus	8	0		
De la Garza Ortiz, F. L. (2003). Identificación de la Presencia del Virus Sin Nombre en Roedores Silvestres del Noreste de México (Hantavirus: Bunyaviridae). Universidad Autónoma de Nuevo León.	Thesis	Nuevo León	*Baiomys taylori*	1	0	ELISA	SNV
			Chaetodipus hispidus	2	0		
			Chaetodipus penicillatus	17	0		
			Dipodomys merriami	4	0		
			Liomys irroratus	1	0		
			Mus musculus	21	0		
			Neotoma albigula	7	0		
			Neotoma mexicana	1	0		
			Neotoma micropus	16	0		
			Onychomys leucogaster	4	0		
			Oryzomys couesi	3	0		

			Perognathus flavus	1	0		
			Peromyscus eremicus	7	0		
			Peromyscus leucopus	46	2		
			Peromyscus levipes	11	2		
			Peromyscus maniculatus	7	0		
			Peromyscus pectoralis	19	2		
			Rattus rattus	2	0		
			Sigmodon hispidus	15	2		
		Tamaulipas	*Chaetodipus hispidus*	7	0	ELISA	SNV
			Mus musculus	1	0		
			Neotoma micropus	13	0		
			Onychomys leucogaster	5	0		
			Peromyscus leucopus	12	0		
			Sigmodon hispidus	4	0		
Chu, Y.-K., Owen, R. D., Sánchez-Hernández, C., Romero-Almaraz, M. de L., & Jonsson, C. B. (2008). Genetic characterization and phylogeny of a hantavirus from Western Mexico. Virus Research, 131(2), 180–188. http://doi.org/10.1016/j.virusres.2007.09.007	Article	Colima	*Liomys pictus*	18	0	IFA	SNV
			Liomys spectabilis	6	0		
			Baiomys musculus	77	1		
			Osgoodomys banderanus	9	0		
			Peromyscus perfulvus	16	0		
			Reithrodontomys fulvescens	18	0		
			Oryzomys couesi	358	23		
			Sigmodon mascotensis	87	6		
			Nyctomys sumichrasti	1	0		
Castro-Arellano, I., Suzán, G., León, R. F., Jiménez, R. M., & Lacher, T. E. (2009). Survey for antibody to hantaviruses in Tamaulipas, Mexico. Journal of Wildlife Diseases, 45(1), 207–212. http://doi.org/10.7589/0090-3558-45.1.207	Article	Tamaulipas	*Peromyscus pectoralis*	62	0	ELISA	SNV
			Sigmodon toltecus	34	0		
			Oryzomys couesi	21	0		
			Liomys irroratus	13	0		
			Baiomys taylori	9	0		
			Oligoryzomys fulvescens	6	0		
			Rattus rattus	3	0		
			Reithrodontomys fulvescens	2	0		
			Mus musculus	1	0		
			Oryzomys rostratus	1	0		
			Peromyscus levipes	31	7		
			Peromyscus ochraventer	11	0		

			Oryzomys chapmani	4	0		
		Chiapas	*Oryzomys couesi*	7	1	ELISA	CADV
			Baiomys musculus	6	1		
		Guerrero	*Baiomys musculus*	1	0	ELISA	CADV
			Peromyscus levipes	2	0		
			Peromyscus megalops	29	1		
			Reithrodontomys sumichrasti	18	3		
		Jalisco	*Baiomys taylori*	13	1	ELISA	CADV
			Peromyscus spicilegus	9	2		
		México	*Peromyscus hylocetes*	8	2	ELISA	CADV
			Peromyscus levipes	1	0		
			Peromyscus melanotis	9	2		
		Michoacan	*Peromyscus melanotis*	1	0	ELISA	CADV
			Peromyscus spp	1	0		
			Reithrodontomys microdon	1	1		
			Reithrodontomys sumichrasti	2	2		
Milazzo, M. L., Cajimat, M. N. B., Romo, H. E., Estrada-Franco, J. G., Ignacio Iñiguez-Dávalos, L., Bradley, R. D., & Fulhorst, C. F. (2012). Geographic distribution of hantaviruses associated with Neotomine and Sigmodontine rodents, Mexico. Emerging Infectious Diseases, 18(4), 571–576. http://doi.org/10.3201/eid1804.111028	Article	Nayarit	*Peromyscus spp*	27	1	ELISA	CADV
		Nuevo León	*Peromyscus eremicus*	5	1	ELISA	CADV
			Peromyscus leucopus	1	0		
			Peromyscus maniculatus	9	1		
			Reithrodontomys megalotis	3	0		
			Peromyscus levipes	21	3		
			Reithrodontomys megalotis	1	0		
		San Luís Potosi	*Peromyscus eremicus*	6	0	ELISA	CADV
			Peromyscus maniculatus	5	1		
			Reithrodontomys megalotis	5	0		
			Peromyscus levipes	16	1		
			Peromyscus ochraventer	11	2		
		Tamaulipas	*Peromyscus leucopus*	7	1	ELISA	CADV
			Baiomys taylori	1	0		
			Peromyscus leucopus	6	1		
		Veracruz	*Peromyscus maniculatus*	4	0	ELISA	CADV
			Peromyscus melanotis	2	0		
			Reithrodontomys megalotis	8	3		
			Baiomys musculus	1	0		

	Peromyscus maniculatus	1	1		
	Peromyscus melanotis	23	0		
	Reithrodontomys megalotis	9	1		
	Peromyscus melanotis	34	1		
	Reithrodontomys megalotis	3	1		
Chihuahua	*Baiomys taylori*	2	0	ELISA	CADV
	Peromyscus boylii	3	0		
	Peromyscus truei	1	0		
	Peromyscus spp	1	0		
	Reitrhodontomys fulvescens	1	0		
	Sigmodon ochrognathus	1	0		
Coahuila	*Peromyscus eremicus*	8	0	ELISA	CADV
	Peromyscus hooperi	6	0		
	Peromyscus pectoralis	2	0		
Guanajuato	*Baiomys taylori*	1	0	ELISA	CADV
	Peromyscus melanophrys	5	0		
	Sigmodon mascotensis	2	0		
Oaxaca	*Baiomys musculus*	14	0	ELISA	CADV
	Habromys ixtlani	5	0		
	Hodomys alleni	1	0		
	Neotoma mexicana	3	0		
	Oryzomys alfaroi	1	0		
	Oryzomys couesi	1	0		
	Peromyscus aztecus	1	0		
	Peromyscus batae	21	0		
	Peromyscus difficilis	1	0		
	Reitrhodontomys fulvescens	1	0		
	Reithrodontomys microdon	1	0		
	Sigmodon mascotensis	14	0		
Puebla	*Baiomys taylori*	4	0	ELISA	CADV
	Peromyscus gratus	5	0		
	Peromyscus melanophrys	5	0		
	Reitrhodontomys fulvescens	1	0		
Sinaloa	*Baiomys taylori*	9	0	ELISA	CADV
Sonora	*Neotoma albigula*	6	0	ELISA	CADV

			Onychomys torridus	3	0		
			Peromyscus boylii	6	0		
			Peromyscus merriami	6	0		
			Peromyscus schmidlyi	1	0		
		Tlaxcala	*Peromyscus difficilis*	8	0	ELISA	CADV
			Peromyscus maniculatus	1	0		
			Reithrodontomis megalotis	7	0		
Kariwa, H., Yoshida, H., Sánchez-Hernández, C., Romero-Almaraz, M. D. L., Almazán-Catalán, J. A., Ramos, C., … Takashima, I. (2012). Genetic diversity of hantaviruses in Mexico: Identification of three novel hantaviruses from Neotominae rodents. Virus Research, 163(2), 486–494. http://doi.org/10.1016/j.virusres.2011.11.013<br><br>Saasa, N., Sánchez-Hernández, C., de Lourdes Romero-Almaraz, M., Guerrero-Ibarra, E., Almazán-Catalán, A., Yoshida, H., … Kariwa, H. (2012). Ecology of hantaviruses in Mexico: Genetic identification of rodent host species and spillover infection. Virus Research, 168(1–2), 88–96. http://doi.org/10.1016/j.virusres.2012.06.020	Article	Morelos	*Heteromys irruratus*	2	0	ELISA	SNV & MTNV
			Microtus mexicanus	15	0		
			Mus musculus	12	0		
			Neotoma alstoni	2	0		
			Neotoma mexicana	1	0		
			Peromyscus hylocetes	13	0		
			Peromyscus maniculatus	2	0		
			Reithrodontomys megalotis	25	2		
		Guerrero	*Baiomys musculus*	5	0	ELISA	SNV & MTNV
			Heteromys irruratus	13	0		
			Heteromys pictus	5	0		
			Hodomys alleni	4	0		
			Megadontomys thomasi	9	1		
			Neotoma picta	6	1		
			Handleyomys melanotis	2	0		
			Oryzomys couesi	16	0		
			Handleyomys alfaroi	3	0		
			Osgoodomys banderanus	36	0		
			Peromyscus mexicanus	10	0		
			Peromyscus aztecus	1	1		
			Peromyscus batae	127	31		
			Peromyscus lepturus	5	0		
			Peromyscus levipes	11	0		
			Peromyscus megalops	41	1		
			Peromyscus melanocarpus	2	0		
			Peromyscus melanophrys	9	0		
			Peromyscus spp	1	0		

			Reithrodontomys spp	1	0		
			Reithrodontomys sumichrasti	15	6		
			Sigmodon mascotensis	1	0		
			Tylomys nudicaudus	11	0		
Arellano, E., Castro-Arellano, I., Suzán, G., González-Cózatl, F. X., & Jiménez, R. M. (2012). Antibody Seroprevalence to Hantaviruses in Rodents from Reserva De La Biosfera Sierra De Huautla, Morelos. Western North American Naturalist, 72(1), 105–109. http://doi.org/10.3398/064.072.0114	Article	Morelos	*Liomys irroratus*	91	1	ELISA	SNV
			Baiomys musculus	24	0		
			Peromyscus levipes	2	0		
			Peromyscus melanophrys	12	0		
			Reithrodontomys fulvescens	19	0		
			Sigmodon hispidus	3	0		
González-Padrón, S. K. (2014). Diversidad y abundancia de roedores reservorios de hantavirus en un gradiente de impacto antropogénico en México. Universidad Nacional Autónoma de México.	Thesis	Veracruz	*Microtus quasiater*	3	0	ELISA	SNV
			Mus musculus	14	1		
			Oryzomys couesi	4	0		
			Peromyscus furvus	23	1		
			Peromyscus leucopus	2	0		
			Peromyscus levipes	6	1		
			Peromyscus maniculatus	28	1		
			Peromyscus mexicanus	15	0		
			Rattus rattus	1	0		
			Reithrodontomys fulvescens	1	0		
			Reithrodontomys mexicanus	1	0		
			Sigmodon hispidus	1	0		
		Hidalgo	*Baiomys taylori*	14	4	ELISA	SNV
			Liomys irroratus	14	2		
			Mus musculus	5	3		
			Perognathus flavus	2	1		
			Peromyscus difficilis	29	11		
			Peromyscus gratus	21	13		
			Peromyscus leucopus	2	1		
			Peromyscus maniculatus	42	28		
			Peromyscus spp	6	1		
			Rattus spp	1	1		
		Campeche	*Heteromys guameri*	7	0	ELISA	SNV
			Ototylomys phyllotis	1	0		

			Peromyscus spp	2	0		
			Rattus rattus	3	0		
			Reithrodontomys gracilis	1	0		
			Sigmodon hispidus	13	1		
		Jalisco	*Liomys pictus*	13	0	ELISA	SNV
			Peromyscus banderanus	14	2		
			Peromyscus perfulvus	14	4		
			Reitrhodontomys fulvescens	1	0		
			Sigmodon mascotensis	1	0		
Moreno-Torres, K., Gual-Sill, F., Morales-Jiménez, R., Rubio, A. V., Ceballos, G., & Suzán, G. (2014). Serological Survey of Hantavirus In Rodents From Prairie Dog Ecosystems In Chihuahua, Mexico. The Southwestern Naturalist, 59(July 2015), 590–594. http://doi.org/10.1894/SGM-37.1	Article	Chihuahua	*Dipodomys merriami*	29	0	ELISA	CADV
			Peromyscus maniculatus	26	3		
			Sigmodon hispidus	9	0		
			Neotoma albigula	9	0		
			Onychomys leucogaster	22	0		
			Perognathus flavus	16	0		
			Dipodomys ordii	3	0		
			Dipodomys spectabilis	7	0		
			Peromyscus leucopus	1	0		
			Reithrodontomys megalotis	1	0		
			Baiomys taylori	26	0		
Ortiz Chabolla, D. J. Efecto Del Paisaje Sobre La Diversidad de Roedores y Prevalencia de Anticuerpos Contra Hantavirus En Una Región Ganadera Del Este de Yucatán, México, Universidad Nacional Autónoma de México, 2018	Thesis	Yucatán	*Heteromys desmarestianus*	7	0	ELISA	SNV
			Heteromys gaumeri	53	3		
			Mus musculus	51	0		
			Oligoryzomys fulvescens	22	2		
			Oryzomys couesi	4	1		
			Ototylomys phyllotis	20	0		
			Peromyscus leucopus	39	2		
			Peromyscus yucatanicus	46	2		
			Rattus rattus	10	2		
			Reithrodontomys gracilis	19	0		
			Sigmodon hispidus	82	13		
Rubio, A. V., Vigueras-Galván, A. L., Schountz, T., Moreno-Torres, K., List, R., Sarmiento-Silva, R. E., … Suzán, G. (2015). Abundance of hantavirus hosts in a landscape with black-tailed prairie dog colonies in	Article	Chihuahua	*Baiomys taylori*	3	2	ELISA	SNV
			Chaetodipus hispidus	22	1		
			Chaetodipus penicillatus	86	11		

northwestern Mexico. Mammalian Biology - Zeitschrift Für Säugetierkunde, 80(6), 491–495. http://doi.org/10.1016/j.mambio.2015.06.004			*Dipodomys merriami*	263	54		
			Dipodomys ordii	9	0		
			Dipodomys spectabilis	114	27		
			Neotoma albigula	8	4		
			Onychomys arenicola	92	20		
			Onychomys leucogaster	13	2		
			Perognathus flavus	42	5		
			Peromyscus boylii	6	1		
			Peromyscus leucopus	18	6		
			Peromyscus maniculatus	46	11		
			Xerospermophilus spilosoma	9	1		
Laboratorio de Ecología de Enfermedades y Una Salud (Laboratory of Disease Ecology and One Health) of the FMVZ-UNAM	Material not intended for publication	Hidalgo	*Peromyscus melanotis*	16	0	ELISA	SNV
			Peromyscus leucopus	1	0		
			Peromyscus levipes	4	0		
			Microtus mexicanus	3	0		
			Reithrodontomys mexicanus	3	0		
			Peromyscus mexicanus	1	0		
			Liomys irroratus	17	0		
			Reithrodontomys mexicanus	10	0		
			Peromyscus maniculatus	2	0		
			Mus musculus	1	0		
			Rattus rattus	1	0		
			Peromyscus melanotis	20	0		
			Peromyscus difficilis	2	0		
			Reithrodontomys megalotis	1	0		
			Liomys irroratus	1	0		
			Peromyscus difficilis	1	0		
		Mexico City	*Peromyscus gratus*	14	6	ELISA	SNV
			Peromyscus difficilis	1	0		
			Mus musculus	4	0		
			Peromyscus melanotis	1	1		
			Mus musculus	2	0		
			Peromyscus melanophrys	1	0		
		Sonora	*Dipodomys merriami*	1	0	ELISA	SNV

	Onychomys arenicola	4	4
	Chaetodipus penicillatus	6	0
	Peromyscus maniculatus	2	1
	Sigmodon hispidus	3	1
	Chaetodipus hispidus	2	0
	TOTAL	3862	392

ELISA: Enzyme-Linked Immunosorbent Assay. IFA: Immunofluorescence Assay. The Laboratorio de Ecología de Enfermedades y Una Salud of the FMVZ-UNAM collected the unpublished data with different sampling designs, all samples obtained by retro-orbital plexus puncture, preserved in Nobuto blood filter strips (Cole-Parmer, Vernon Hills,IL) and analyzed in the Laboratorio de Virología of the FMVZ-UNAM.

Supplementary table S2. Seroprevalence by State

State	Analyzed	Positives	SP(%)	WSP (%)
Campeche	27	1	3.70	5.30
Chiapas	13	2	15.38	17.14
Chihuahua	880	147	16.70	49.19
Coahuila	19	0	-	-
Colima	590	20	3.39	9.39
Mexico City	33	9	27.27	41.41
Mexico State	35	8	22.86	35.29
Guanajuato	8	0	-	-
Guerrero	384	45	11.72	30.29
Hidalgo	220	65	29.55	69.21
Jalisco	88	9	10.23	19.89
Michoacan	5	3	6-	41.94
Morelos	223	3	1.35	3.16
Nayarit	27	1	3.70	5.30
Nuevo Leon	225	13	5.78	13.59
Oaxaca	70	0	-	-
Puebla	15	0	-	-
San Luis Potosi	43	4	9.30	15.20
Sinaloa	9	0	-	-
Sonora	40	6	15.00	24.03
Tamaulipas	254	9	3.54	8.52
Tlaxcala	16	0	-	-
Veracruz	184	11	5.98	13.54
Yucatan	445	25	5.62	14.88

SP. Seroprevalence. WSP. Weighted seroprevalence

Suplementary table S3. Seroprevalence by species

Family	Species	Analyzed	Positives	SP (%)	WSP
Cricetidae	*Baiomys musculus*	130	2	1.54	3.25
Cricetidae	*Baiomys taylori*	83	7	8.43	16.18
Cricetidae	*Habromys ixtlani*	5	0	-	-
Cricetidae	*Handleyomys alfaroi*	3	0	-	-
Cricetidae	*Handleyomys melanotis*	2	0	-	-
Cricetidae	*Hodomys alleni*	1	0	-	-
Cricetidae	*Megadontomys thomasi*	9	1	11.11	10.60
Cricetidae	*Microtus mexicanus*	19	0	-	-
Cricetidae	*Microtus quasiater*	3	0	-	-
Cricetidae	*Neotoma albigula*	30	4	13.33	19.69
Cricetidae	*Neotoma alstoni*	2	0	-	-
Cricetidae	*Neotoma mexicana*	5	0	-	-
Cricetidae	*Neotoma micropus*	29	0	-	-
Cricetidae	*Neotoma picta*	6	1	16.67	12.97
Cricetidae	*Nyctomys sumichrasti*	1	0	-	-
Cricetidae	*Oligoryzomys fulvescens*	29	2	6.90	10.09
Cricetidae	*Onychomys arenicola*	96	24	25.00	49.56
Cricetidae	*Onychomys leucogaster*	44	2	4.55	7.47
Cricetidae	*Onychomys torridus*	3	0	-	-
Cricetidae	*Oryzomys alfaroi*	1	0	-	-
Cricetidae	*Oryzomys chapmani*	4	0	-	-
Cricetidae	*Oryzomys couesi*	414	25	6.04	15.80
Cricetidae	*Oryzomys palustris*	1	0	-	-
Cricetidae	*Oryzomys rostratus*	1	0	-	-
Cricetidae	*Osgoodomys banderanus*	51	0	-	-
Cricetidae	*Ototylomys phyllotis*	21	0	-	-
Cricetidae	*Peromyscus aztecus*	2	1	50	15.05
Cricetidae	*Peromyscus banderanus*	14	2	14.29	16.37
Cricetidae	*Peromyscus beatae*	148	31	20.95	45.46
Cricetidae	*Peromyscus boylii*	15	1	6.67	7.84
Cricetidae	*Peromyscus difficilis*	42	11	26.19	42.51
Cricetidae	*Peromyscus eremicus*	27	1	3.70	5.30
Cricetidae	*Peromyscus furvus*	23	1	4.35	5.92
Cricetidae	*Peromyscus gratus*	40	19	47.50	76.10
Cricetidae	*Peromyscus hooperi*	6	0	-	-
Cricetidae	*Peromyscus hylocetes*	29	4	13.79	20.17
Cricetidae	*Peromyscus lepturus*	5	0	-	-
Cricetidae	*Peromyscus leucopus*	133	13	9.77	20.76
Cricetidae	*Peromyscus levipes*	107	14	13.08	26.55
Cricetidae	*Peromyscus maniculatus*	183	48	26.23	59.34
Cricetidae	*Peromyscus megalops*	70	2	2.86	5.27
Cricetidae	*Peromyscus melanocarpus*	2	0	-	-
Cricetidae	*Peromyscus melanophrys*	32	0	-	-
Cricetidae	*Peromyscus melanotis*	115	6	5.22	10.75
Cricetidae	*Peromyscus merriami*	6	0	-	-
Cricetidae	*Peromyscus mexicanus*	26	0	-	-

Cricetidae	*Peromyscus ochraventer*	22	2	9.09	12.20
Cricetidae	*Peromyscus pectoralis*	83	2	2.41	4.62
Cricetidae	*Peromyscus perfulvus*	30	4	13.33	19.69
Cricetidae	*Peromyscus schmidlyi*	1	0	-	-
Cricetidae	*Peromyscus spicilegus*	9	2	22.22	21.21
Cricetidae	*Peromyscus spp*	38	2	5.26	8.31
Cricetidae	*Peromyscus truei*	1	0	-	-
Cricetidae	*Peromyscus yucatanicus*	115	2	1.74	3.58
Cricetidae	*Reithrodontomys fulvescens*	44	0	-	-
Cricetidae	*Reithrodontomys gracilis*	22	0	-	-
Cricetidae	*Reithrodontomys megalotis*	69	7	10.14	18.65
Cricetidae	*Reithrodontomys mexicanus*	14	0	-	-
Cricetidae	*Reithrodontomys spp*	1	0	-	-
Cricetidae	*Reithrodontomys sumichrasti*	36	12	33.33	51.88
Cricetidae	*Reitrodontomys microdon*	2	1	50	15.05
Cricetidae	*Sigmodon hispidus*	138	17	12.32	26.36
Cricetidae	*Sigmodon mascotensis*	105	6	5.71	11.55
Cricetidae	*Sigmodon ochrognathus*	1	0	-	-
Cricetidae	*Sigmodon toltecus*	34	0	-	-
Cricetidae	*Tylomys nudicaudus*	11	0	-	-
Heteromydae	*Chaetodipus hispidus*	33	1	3.03	4.60
Heteromydae	*Chaetodipus penicilatus*	109	11	10.09	20.56
Heteromydae	*Dipodomys merriami*	299	54	18.06	44.71
Heteromydae	*Dipodomys ordii*	12	0	-	-
Heteromydae	*Dipodomys spectabilis*	121	27	22.31	46.48
Heteromydae	*Heteromys desmarestianus*	7	0	-	-
Heteromydae	*Heteromys guameri*	60	3	5.00	8.89
Heteromydae	*Heteromys irruratus*	15	0	-	-
Heteromydae	*Heteromys pictus*	5	0	-	-
Heteromydae	*Hodomys alleni*	4	0	-	-
Heteromydae	*Liomys irroratus*	137	3	2.19	4.68
Heteromydae	*Liomys pictus*	45	0	-	-
Heteromydae	*Liomys spectabilis*	6	0	-	-
Heteromydae	*Perognatus flavus*	61	6	9.84	17.56
Muridae	*Mus musculus*	112	4	3.57	7.32
Muridae	*Rattus rattus*	32	2	6.25	9.41
Muridae	*Rattus spp*	1	1	100	-

SP. Seroprevalence. WSP. Weighted seroprevalence
